# Spatial and temporal determinants of genetic structure in *Gentianella bohemica*

**DOI:** 10.1002/ece3.211

**Published:** 2012-03

**Authors:** Julia Königer, Carolin A Rebernig, Jiří Brabec, Kathrin Kiehl, Josef Greimler

**Affiliations:** 1 Faculty of Agricultural Sciences and Landscape Architecture, University of Applied Sciences OsnabrückOldenburger Landstraße 24, D-49090 Osnabrück, Germany; 2 Department of Plant Biology and Forest Genetics, Swedish University of Agricultural SciencesUppsala BioCenter, SE-75007 Uppsala, Sweden; 3 Muzeum ChebKrále Jiřího z Poděbrad 493/4, 350 11 Cheb, Czech Republic; 4 Department of Systematic and Evolutionary Botany, University of ViennaRennweg 14, A-1030 Vienna, Austria

**Keywords:** AFLP, biennial, bottleneck, effective population size, genetic diversity, habitat fragmentation, historical distribution, isolation, spatial patterns

## Abstract

The biennial plant *Gentianella bohemica* is a subendemic of the Bohemian Massif, where it occurs in seminatural grasslands. It has become rare in recent decades as a result of profound changes in land use. Using amplified fragment length polymorphisms (AFLP) fingerprint data, we investigated the genetic structure within and among populations of *G. bohemica* in Bavaria, the Czech Republic, and the Austrian border region. The aim of our study was (1) to analyze the genetic structure among populations and to discuss these findings in the context of present and historical patterns of connectivity and isolation of populations, (2) to analyze genetic structure among consecutive generations (cohorts of two consecutive years), and (3) to investigate relationships between intrapopulational diversity and effective population size (*N*_e_) as well as plant traits. (1) The German populations were strongly isolated from each other (pairwise *F*_ST_= 0.29–0.60) and from all other populations (*F*_ST_= 0.24–0.49). We found a pattern of near panmixis among the latter (*F*_ST_= 0.15–0.35) with geographical distance explaining only 8% of the genetic variance. These results were congruent with a principal coordinate analysis (PCoA) and analysis using STRUCTURE to identify genetically coherent groups. These findings are in line with the strong physical barrier and historical constraints, resulting in separation of the German populations from the others. (2) We found pronounced genetic differences between consecutive cohorts of the German populations (pairwise *F*_ST_= 0.23 and 0.31), which can be explained by local population history (land use, disturbance). (3) Genetic diversity within populations (Shannon index, *H*_Sh_) was significantly correlated with *N*_e_ (*R*_S_= 0.733) and reflected a loss of diversity due to several demographic bottlenecks. Overall, we found that the genetic structure in *G. bohemica* is strongly influenced by historical periods of high connectivity and isolation as well as by marked demographic fluctuations in declining populations.

## Introduction

Comparing historical and contemporary data on the distribution of plant species of seminatural grasslands often provides a picture of dramatic loss of populations, of decreasing population size, and of increasing habitat fragmentation. This is chiefly due to extrinsic factors such as habitat alteration and destruction, land-use intensification (or the abandonment of traditional land use) in seminatural habitats, climate change, and the interaction between these threats (Pfeiffer and Jetschke 2006; Tylianakis et al. 2008; De Chazal and Rounsevell 2009; Pautasso et al. 2010). In Europe, this process started about 100–150 years ago, causing massive habitat loss and modification of species-rich poorly productive grasslands (e.g., Eriksson et al. 2002; Poschlod and Wallis De Vries 2002; Baessler et al. 2010). The disintegration of large habitats into small isolated patches reduced the population sizes of formerly frequent plant species tremendously, and with increasing geographical distance between suitable habitat patches, gene flow between populations was hampered or even stopped. The loss of species diversity is the final consequence of the dramatic reduction and fragmentation of these habitat types, and is especially pronounced over the last few decades (Beaufoy et al. 1995; Fischer and Stöcklin 1997; Van Andel and Aronson 2006).

In fragmented habitats, short-lived plant species with high generational turnover often experience additional pressure from intrinsic factors connected to their life cycle, which renders them more sensitive to environmental stochasticity, especially when they lack a persistent seed bank (Matthies et al. 2004). They may respond rapidly to decreases in habitat size by forming smaller populations (Kiviniemi 2008), which over time affects genetic structure and diversity. In general, smaller population sizes and isolation can result in reduced fitness (Ellstrand and Elram 1993; Oostermeijer et al. 1994; Fischer and Matthies 1998a; Lienert et al. 2002) and/or reduced genetic diversity (Fischer and Matthies 1997; Leimu et al 2006; Jacquemyn et al. 2009a). In contrast, such effects can be delayed in populations of long-lived perennial plants (Young et al. 1996; Jacquemyn et al. 2007; Gaudeul and Till–Bottraud 2008).

Until now, the historical dimension in population genetics has mostly been studied in terms of phylogeography over large regional scales and over long time periods, for example, Pleistocene history, reviewed by Tribsch and Schönswetter (2003) and Schönswetter et al. (2005). In contrast, studies at local or regional scales have focused on ecological, spatial, management, and intrinsic determinants (e.g., breeding system, reproduction) of population structure. Studies addressing temporal variation of the genetic structure at a population level have focused for instance on variation between age classes in perennial herbs (Jacquemyn et al. 2009b), in woody plants (e.g., Chung et al. 2003; Kelly et al. 2004), or on variation among seasonal cohorts of ephemerals (Haldimann et al. 2003). Variation between two generations separated by four years in the biennial *Gentianella aspera* was investigated by Stadler et al. (2010). To our knowledge, no other studies on genetic differentiation among cohorts in biennial plants exist.

**Figure 1:**
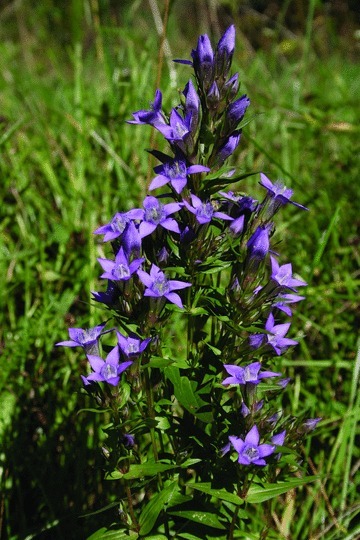
*Gentianella bohemica* in a meadow near the Czech–German border.

**Figure 2:**
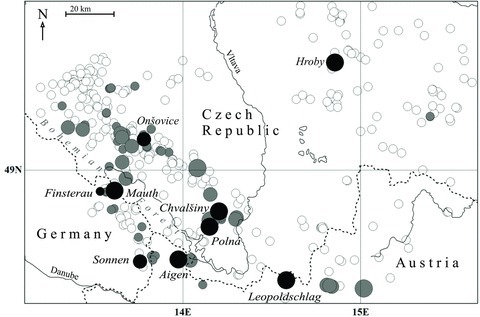
Study area showing the investigated populations (black), the distribution of recent (gray) and extinct populations (hollow circles). Circle sizes for recent and investigated populations correspond to three size classes according to the effective population size *N*_e_ (small: <10, intermediate: <50, large: >100).

*Gentianella bohemica* Skalicky (*G. praecox* A. and J. Kerner subsp. *bohemica* [Skalicky] Holub) is a biennial plant that has become rare during the 20th century (Fig. 1). Before the Second World War, the species was wide spread in the Bohemian Massif (Fig. 2) and was so common that it was collected to cure mastitis in cattle (Berg 2001). Recently, however, it has shown a dramatic decline through afforestation, land-use abandonment and intensified agriculture (Berg 2001; Rösler 2001; Brabec 2005; Engleder 2006; Dolek et al. 2010). Dispersal vectors such as cattle drives or the harvesting and transport of hay are largely absent nowadays. Apart from a few Czech and Austrian populations containing thousands of individuals each (Brabec 2005; Engleder 2006), many of the extant populations are small and isolated and frequent transfer of diaspores or pollen between populations is unlikely. Despite the introduction of conservation measures in the 1990s, many populations have not recovered significantly (Dolek et al. 2010). *Gentianella bohemica* is highly adapted to traditional land use and seems to be very sensitive to changes in land use, similar to other biennial *Gentianella* species in Europe (Pritchard 1972; Greimler and Dobeš 2000; Lennartsson 2000; Korneck et al. 1996).

The aim of this study was to identify the relationships between the genetic structure of *G. bohemica* populations and data on local and regional population history, focusing on Bavaria, southwest Bohemia, and the Austrian border region. We used DNA amplified fragment length polymorphisms (AFLP; Vos et al. 1995) to analyze: (1) genetic structure among populations and discuss these results in the context of present and historical patterns of connectivity and isolation among populations; (2) genetic structure among consecutive generations, that is, cohorts of the years 2007 and 2008 in a subset of those populations; (3) diversity within populations and its relationships with population size and plant traits.

## Material and Methods

### Study species

*Gentianella bohemica* is a subendemic species of the Bohemian Massif, which grows mainly in montane, traditionally grazed or mown *Nardus* grasslands on siliceous substrates (Rösler 2001; Engleder 2006; Dolek et al. 2010; Fig. 1). It also grows to a lesser extent in mesic or wet meadows (Arrhenatherion and Molinion), and also in dry calcareous grasslands and pastures (Bromion erecti and Koelerio-Phleion phleoidis) in parts of Bohemia (Brabec 2005). At present, it is found mainly in the Czech Republic and Austria, with only a few scattered populations in Germany and Poland. Most of these populations seem to be isolated by distances of several kilometers. Population sizes of this biennial species fluctuate considerably between years (Rösler 2001; Brabec 2005; Engleder 2006). The most frequently observed pollinators are *Apis mellifera* (honeybee) and *Bombus* spp. (bumblebees). Other pollinators are *Lepidoptera* (butterflies), *Syrphidae* (hoverflies), and *Muscidae* (flies) (Göldel et al. 2010). There is evidence that *G. bohemica* forms a persistent seed bank (Brabec 2010b; J. Brabec, unpubl. data) as seen in the related *G. germanica* (Fischer and Matthies 1998b). *Gentianella bohemica* is strongly protected in Europe and is listed in Annexes II and IV of the Habitats Directive (Council of the European Community 1992). Data for the map showing the recent and historical distribution of *G. bohemica* (Fig. 2) in the Bohemian Massif were taken from the literature (Sendtner 1860; Wettstein 1896; Vollmann 1914; Graf 1938; Deyl 1973; Čížek and Král 1974; Götz 1990; Skalický and Sofron 1990; Pavlíčko 1999; Kirschner and Kirschnerová 2000; Rösler 2002; Pavlíčko 2008; Zipp 2009; Brabec 2010b; Engleder 2010), the herbaria BRNM, BRNU, CB, GM, GZU, CHEB, LI, LIM, LIT, M, MJ, MP, OLM, OP, PL, PR, PRC, ROZ, SOB, W, WU, the Bavarian Forest National Park Authority in Grafenau, and personal observations. Because the current taxonomic concept of *G. bohemica* was only fixed in 1969 (Skalický 1969), we have included all earlier literature records of *G. austriaca* from the Bohemian Massif. This agrees with the distribution pattern gained from herbarium vouchers revised for the map. Size classes of recent populations are based on effective population size *N*_e_ using census data from regional monitoring programs (Rösler 2002; Brabec 2010b; Engleder 2010), mostly from census data of the last 10–20 years.

### Plant material and population data

In 2008, leaf samples were collected from 10 populations of *G. bohemica* in the Czech Republic, Austria, and Germany, and one population of unclear taxonomic status (*G. germanica/lutescens*) near Dresden, Germany. Between 25 (exceptionally eight) and 33 individuals per population were selected randomly. Additional leaf samples, collected in 2007 from a subset of four *G. bohemica* populations, were also included. Samples were dried and stored in silica gel prior to DNA isolation. Vouchers of the sampled populations are stored in the herbaria CHEB, LI, PR, and REG. For each population, census data (number of flowering plants) were available from regional monitoring programs with census periods ranging from seven to 20 consecutive years (Appendix S1). Geographic distances between the investigated populations ranged from 2.5 to ca. 100 km. Data on plant traits (shoot length, number of internodes, and number of flowers) were collected in the field from 30 adult individuals per population (eight individuals in the small Finsterau population), except for the populations at Aigen and Leopoldschlag, for which permission was not received.

### Amplified Fragment Length Polymorphisms

Total genomic DNA from silica gel-dried leaf material was extracted using DNeasy 96 Plant Kit (Qiagen, Hilden, Germany) according to the manufacturer's protocol. AFLP fingerprint profiles were generated for (exceptionally 8) 25–33 individuals per population, totaling 253 individuals, following the protocol described in Dixon et al. (2008). Two negative controls were included in each PCR and 6.25% of the samples were replicated. Three primer combinations were selected for the final analyses (Jang et al. 2005; fluorescent dyes in brackets): EcoRI-ATC/MseI-CTC (FAM), EcoRI-AGG/MseI-CAC (VIC), EcoRI-AGC/MseI-CAC (NED). Selective PCR products were purified using Sephadex G-50 Superfine (GE Healthcare Bio-Sciences, Uppsala, Sweden) according to the manufacturer's instructions. Amplified products were run on a 3130xl Genetic Analyzer capillary sequencer (Applied Biosystems, Foster City, CA) with GeneScan 500 ROX as the internal size standard (Applied Biosystems). Raw data were aligned with the internal size standard using ABI Prism GeneScan 3.7.1 (Applied Biosystems). Subsequently, the GeneScan files were imported into Genographer (ver. 1.6.0; http://hordeum.oscs.montana.edu/genographer) for scoring. Bands in the size range 100–500 bp were scored and the results were exported as a presence/absence matrix. We chose not to score fragments below a length of 100 bp due to the higher frequency of nonhomologous fragments in this size class (Vekemans et al. 2002). Nonreproducible bands identified by comparisons among replicated individuals were excluded from further analyses. Across the 253 individuals, the number of fragments found by the three primer combinations were 212 (FAM), 143 (VIC), and 119 (NED), of which 5, 11, and 1, respectively, were not reproducible and were therefore excluded from the matrix, resulting in a total of 457 fragments and an overall error rate of 3.59%. After initial screening of the matrix we removed further samples for two reasons: (1) As it was impossible to clarify the taxonomic status of the *G. germanica/lutescens* samples until the end of this study, those samples were not included in further analyses. (2) All samples of one Austrian population of *G. bohemica* were also removed, as we found an extremely high number of private fragments due to an infection. Thus, all further analyses were carried out with a final matrix of 379 fragments of 204 individuals from nine populations of *G. bohemica* (see Table 1; Fig. 2, matrix of all fragments available from corresponding author on request).

**Table 1:** The investigated populations with their habitat characteristics, population sizes in 2007 or 2008 (*N*_07/08_); estimated effective population size due to variable population size (*N*_e_), number of available census years (*N*_C_) for calculating *N*_e_, number of analyzed individuals (*n*), mean number of fragments (*n*_F_), unique (private) fragments (*n*_P_), Shannon diversity index (*H*_sh_) with standard deviation (SD).

Acronym	Population	Country	Habitat	Sampling year	*N*_07/08_	*N*_e_	*N*_C_	*n*	*n*_F_	*n*_P_	*H*_sh_	SD
Fi08	Finsterau near Mauth	D	Violo-Nardion and Molinion grassland, 850 m	2008	16	3	20	4	110	2	6.29	1.66
Fi07				2007	30			12	106	11	6.68	0.75
Ma08	Mauth	D	Violo-Nardion grassland, few *Picea abies* trees, 865 m	2008	92	60	20	16	105	2	7.08	0.63
Ma07				2007	420			15	90	5	6.44	0.56
So08	Sonnen near Breitenberg	D	Violo-Nardion grassland, 830 m	2008	271	14	20	21	115	3	6.81	0.48
So07				2007	368			9	109	2	6.68	0.92
Ch08	Chvalšiny	CZ	Arrhenatherion grassland, partly Bromion erecti, 645 m	2008	1360	447	10	21	99	0	7.14	0.50
Ch07				2007	230			12	94	2	6.86	0.78
Ho08	Hroby near Radenín	CZ	Arrhenatherion und Violion caninae grassland, 510 m	2008	1055	912	10	18	105	7	7.05	0.56
On08	Onšovice near Čkyně	CZ	Bromion erecti grassland, partly forest, 630 m	2008	1108	45	10	15	106	1	6.91	0.62
Po08	Polná na Šumavě near Boletice	CZ	Bromion erecti and Arrhenatherion grassland, 780 m	2008	937	354	7	20	117	8	7.13	0.53
Ai08	Aigen	A	very dry Nardion grassland, 935 m	2008	318	57	16	22	107	4	6.77	0.43
Le08	Leopoldschlag	A	dry Nardion grassland, 830 m	2008	365	63	7	19	114	13	7.14	0.54

A, Austria; CZ, Czech Republic; D, Germany.

### Data analysis

Because of the fluctuating population sizes of biennial species, a proxy of the effective population size *N*_e_ was calculated from the harmonic mean of census sizes of flowering individuals over several years (Hartl and Clark 2007). The number of census years is given in Table 1. Neighbor nets of the whole dataset were constructed with Splits Tree4 v4.10 (Huson and Bryant 2006), using standard settings, including 1000 bootstraps, to investigate the pattern among the AFLP phenotypes of all analyzed populations in 2007 and 2008. Principal coordinate analysis (PCoA) was conducted in order to analyze patterns of similarity using GenAlex 6 (Peakall and Smouse 2006). In this analysis, principal coordinates can be extracted from a squared Euclidean distance matrix. Mantel tests were performed with GenAlex 6 to reveal possible relationships between genetic distances (squared Euclidean distances) and geographic distances (km) among the samples of 2008. This relationship is described by the correlation coefficient (*R*_M_). The significance of the correlations was tested by random permutation (9999 permutations). Two- and three-level hierarchical analyses of molecular variance (AMOVA) were calculated for regional groupings (geographic regions, countries) and temporal groupings (cohorts of consecutive years) using the program Arlequin 3.0 (Excoffier et al. 2005). The significance of the components of the variance was tested with 1023 random permutations. Arlequin 3.0 was also used to calculate molecular diversity within populations (π, the mean number of pairwise differences). Additionally, we calculated the Shannon diversity index (*H*_Sh_) based on polymorphic fragments using the software FAMD 1.25 (Schlüter and Harris 2006). As the two diversity estimates (π and *H*_Sh_) for the smallest population sample (Finsterau 2008, with only four individuals) disagreed markedly, we removed this sample from all correlation analyses. The two diversity estimates then revealed the same pattern of significant correlations and were significantly correlated regardless of whether we included the 2007 samples (nonparametric Spearman's rank correlation coefficient *R*_S_= 0.949, *P* < 0.001) or considered only 2008 samples with Finsterau 2007 replacing 2008 (*R*_S_= 0.967, *P* < 0.001). We therefore only present the results for *H*_Sh_. Correlations (*R*_S_) between indices of genetic diversity, population sizes, and plant traits (pooled for each population) were calculated with STATISTICA 6.0 (StatSoft 2003).

Genetically homogeneous groups of individuals were identified according to the genetic mixture analysis implemented in STRUCTURE 2.2 (Pritchard et al. 2000). The appropriate number of groups (*K*) and the most likely assignment of each individual to a certain group without a priori information about population structure were estimated using models with uncorrelated allele frequencies, with or without admixture. *K* values ranging from 1 to 10 were tested, employing 10 independent runs for each value of *K*, each with 10^6^ Markov chain Monte Carlo generations after a burn-in period of 10^5^ generations. The optimal number of groups was determined from the similarity coefficients among replicated runs of the same *K* as defined by Rosenberg et al. (2002) as implemented in the R script STRUCTURE 2.1-SUM (Ehrich 2006). Results of the replicated runs were averaged using CLUMPP 1.1.1 (Jakobsson and Rosenberg 2007).

## Results

Overall, 379 unambiguously scorable and reproducible fragments with lengths of 100–500 bp were found in 204 individuals of *G. bohemica*. Of these fragments, 371 were polymorphic and 41 of them occurred only in one individual each. Every individual had a different multilocus AFLP phenotype. Details of the mean number of fragments, number of polymorphic fragments, and number of private fragments per population are given in Table 1.

### Genetic structure and spatial context

**Figure 3:**
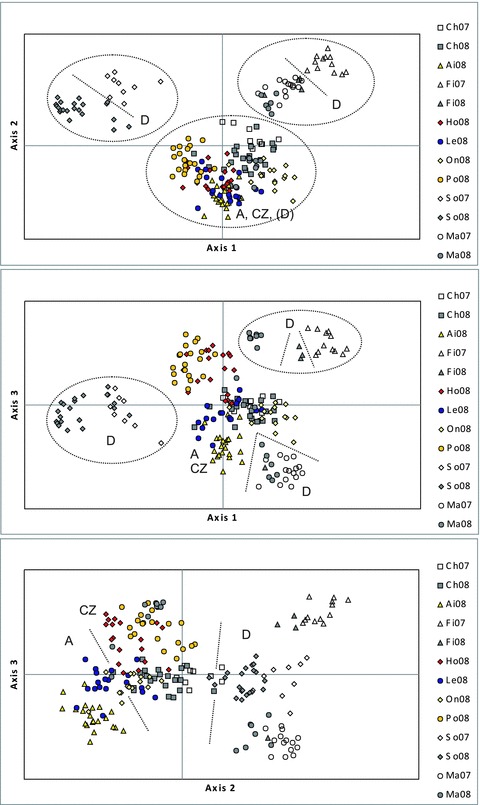
PCoA of all AFLP phenotypes showing all combinations of the first three axes. The German populations are well separated from the Czech/Austrian cluster in all cases. The 2007 and 2008 cohorts of the German populations are separated when axis 1 is combined with axes 2 and 3. Population acronyms as in Table 1. A, CZ, and D in the diagrams indicate Austrian, Czech, and German populations, respectively.

A neighbor-net analysis of all 2007 and 2008 samples (data not shown) resulted in a star-like topology of the entire dataset, with some separation between the German populations. The first three axes of the PCoA explained 64% of the total variation (Fig. 3) and provided further resolution among populations and regional groups. Along the first axis (26.2%), the German population at Sonnen differed strongly from the populations at Finsterau and Mauth. The second axis separated the three German populations from the majority of the Czech and Austrian populations. The Czech and Austrian populations were partly separated along the third axis (15.2%) with lowest values for the population at Aigen (Austria) and highest scores for the population at Polná (Czech Republic).

AMOVA partitioned 30.8% (*P* < 0.001) of the variation among populations in 2008 (Table 2A). Testing for differentiation among regional groups revealed that the highest variation was between the groups of (1) all German populations and (2) all Czech and Austrian populations (5.7%, *P* < 0.001) (Table 2B). Accordingly, pairwise *F*_ST_ (Table 3) among the 2008 cohorts of all populations revealed much lower differentiation among Czech and Austrian populations, ranging from 0.15 to 0.35, while differentiation between them and the three German populations ranged from 0.24 to 0.49. We often found very low pairwise *F*_ST_ between the Czech and Austrian populations despite large geographical distances, for example, 0.16 for Polná/Hroby, which also share five fragments not found elsewhere and 0.17 for Chvalšiny/Leopoldschlag. The greatest differentiation, however, was found among the German populations, ranging from 0.29 to 0.60.

**Table 2:** AMOVA two- and three-level designs and results: (A, B) among all populations 2008; (C, D) among four populations (Chvalšiny, Sonnen, Finsterau, Mauth) 2007 and 2008.

Source of variation	df	Percentage of variation
(A) Among all populations 2008	8	30.78***
Within populations	147	69.22***
(B) Among Bavarian and all other populations 2008	1	5.65***
Among populations within groups	7	27.25***
Within populations	146	67.10***
(C) Among four populations 2007	3	44.59***
Within populations	44	55.41***
(D) Among four populations 2008	3	36.13***
Within populations	58	63.87***

df, degree of freedom;

*** *P* < 0.001.

**Table 3:** Pairwise genetic distances (pairwise *F*_ST_) among four populations/cohorts of 2007 and nine of 2008. ^*^P < 0.05; ^*^^*^P < 0.01; ^*^^*^^*^P < 0.001. *P* -values indicate the probability that a random genetic distance is larger than the observed distance and are based on 1000 permutations. For population acronyms see Table 1.

	Fi08	Fi07	Ma08	Ma07	So08	So07	Ch08	Ch07	Ho08	On08	Po08	Ai08
Fi07	0.30^***^											
Ma08	0.29^*^^*^	0.46^***^										
Ma07	0.38^***^	0.51^***^	0.31^***^									
So08	0.51^***^	0.60^***^	0.43^***^	0.53^***^								
So07	0.50^***^	0.53^***^	0.42^***^	0.49^***^	0.23^***^							
Ch08	0.32^***^	0.41^***^	0.24^***^	0.31^***^	0.38^***^	0.33^***^						
Ch 07	0.36^***^	0.43^***^	0.29^***^	0.33^***^	0.42^***^	0.36^***^	0.03^*^					
Ho08	0.35^***^	0.47^***^	0.28^***^	0.41^***^	0.38^***^	0.40^***^	0.15^***^	0.21^***^				
On08	0.41^***^	0.50^***^	0.29^***^	0.43^***^	0.49^***^	0.47^***^	0.22^***^	0.30^***^	0.29^***^			
Po08	0.31^***^	0.47^***^	0.28^***^	0.42^***^	0.34^***^	0.37^***^	0.21^***^	0.24^***^	0.16^***^	0.31^***^		
Ai08	0.47^***^	0.55^***^	0.37^***^	0.47^***^	0.46^***^	0.47^***^	0.24^***^	0.34^***^	0.30^***^	0.35^***^	0.35^***^	
Le08	0.37^***^	0.48^***^	0.27^***^	0.40^***^	0.40^***^	0.39^***^	0.17^***^	0.23^***^	0.22^***^	0.24^***^	0.23^***^	0.24^***^

**Figure 4:**
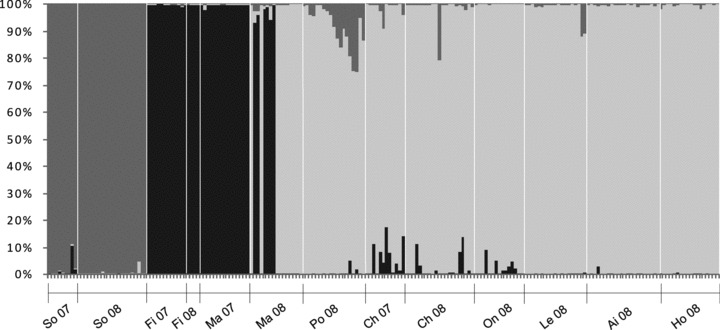
STRUCTURE diagram showing two genetic groups formed by the German populations and a third group comprising all Czech and Austrian populations including a group of individuals (second gene pool) of the German population Ma 08.

Overall, moderate isolation by distance was revealed by a Mantel test (*R*_M_= 0.25; *P* < 0.01) when all populations were compared (2008 cohorts). This isolation by distance remained at the same level (*R*_M_= 0.28; *P* < 0.01), when the German populations were excluded. Geographic distance therefore accounted only for about 6% (*R*^2^= 0.063) of the genetic distance among all populations and for about 8% (*R*^2^= 0.076) of genetic distance among the Czech and Austrian populations. A rigorous analysis of all 2007 and 2008 data for genetically coherent groups using STRUCTURE revealed three groups (Fig. 4). Iterations with *K*= 3 always had the highest similarity coefficient and no variation in the ln p(d) values (Appendix S2). Group 1 included all Czech and Austrian populations and some individuals from Mauth, while group 2 included most individuals from Mauth and Finsterau. The third German population—Sonnen—formed a separate group. Notable proportions of admixture from groups 2 and 3 were found in the populations at Chvalšiny and Polná (Czech Republic).

### Genetic structure in temporal context

The 2007 and 2008 cohorts of the German populations were notably separated in the PCoA, while the two cohorts of the population at Chvalšiny were clustered together in all configurations (Fig. 3). This is reflected in the estimates of pairwise *F*_ST_ with values of 0.23–0.31 (all *P* < 0.001) between the German cohorts and a very low differentiation of 0.03 (*P*< 0.05) between the cohorts of the Czech population. The 2007 and 2008 cohorts were always found in their respective group of the STRUCTURE analysis except for Mauth: the 2007 cohort of this population and part of the 2008 cohort formed a group including the population at Finsterau, located a few kilometers away. The other part of the 2008 cohort was included in the large group formed by all Czech and Austrian populations. The pairwise *F*_ST_ between these two genetic groups of Mauth 2008 is quite high with a value of 0.55. Pairwise *F*_ST_ values between the 2007 cohort and the two groups of 2008 differ notably (0.18 and 0.57, respectively). Due to this complex structure of the 2008 cohort in Mauth, a lower differentiation among all 2008 cohorts was found by AMOVA than among the 2007 cohorts from the same populations (Table 2C and 2D).

A closer inspection of the population from Mauth revealed a lower mean number of fragments (*n*_F_= 90) in the 2007 cohort than in 2008 (*n*_F_= 105), which is in part due to a higher number of low-frequency fragments and fewer fixations in the latter. There are no fixed private fragments in either cohort. Within the 2008 cohort, however, the two private fragments are confined to individuals that are included in the large genetic group of all non-German populations. Regarding this group of individuals, the private fragments occur with high frequencies of 0.7 and 0.8. Both 2008 groups show similar *n*_F_ values (106 for the group joining the large cluster vs. 103).

### Diversity within populations

The Shannon diversity index (*H*_Sh_) did not show any correlation with *N*, the current population size. However, *H*_Sh_ correlated significantly (*R*_S_= 0.612, *P* < 0.05 for all samples and *R*_S_= 0.733, *P* < 0.05 for the 2008 samples) with the estimated effective population size *N*_e_. High *H*_Sh_ values were found for the Czech populations but also in the population at Mauth 2008 (Germany) as well as that at Leopoldschlag (Austria). In contrast, the other Austrian and all other German populations showed lower values than the Czech populations, which also exhibited the highest numbers of individuals in the 2008 census and showed the largest effective population sizes (except for Onšovice). We noted a striking difference in *H*_Sh_ between the 2007 and 2008 cohorts of Mauth. Among the measured plant traits, we found a significant correlation (*R*_S_= 0.644, *P* < 0.05 for all samples and *R*_S_= 0.786, *P* < 0.05 for the 2008 samples) of *H*_Sh_ with shoot length.

## Discussion

### Spatial structure and historical context

In our study, genetic structure provided strong signals of isolation among the three German populations of *G. bohemica* themselves as well as between them and the Czech and Austrian populations. This mirrors historical patterns of connection and regional population history. At the regional scale, there is evidence of a much higher number of populations in the past in the whole region (Fig. 2). Despite the high number of 20 or more former populations (of which only seven small ones have survived) between the population at Mauth (Germany) and Onšovice (the nearest large Czech population), several features could have impeded gene flow between the two. The German *G. bohemica* populations are separated from the Czech populations by the main ridge of the Bohemian Forest (Böhmerwald, Šumava), which is covered by dense spruce forests and forms a barrier to the dispersal of pollen and diaspores. Additionally, wet fen areas, unfavorable for *G. bohemica*, extend across the border region. Interestingly, the genetic structure of the investigated populations indicates a scenario of former panmixis within a well-connected population system in the former Habsburg Empire (covering the regions of today's Austria and Czech Republic) from which the German populations were already disconnected in historical times despite their geographical proximity.

Historical trade routes have provided a variety of vectors facilitating seed dispersal since medieval times. Several ancient pathways existed between Upper Austria and Bohemia for trading in salt and cattle (Hajná 2011). Two of these routes passed the localities of the investigated Austrian populations and continued close to the Czech populations Chvalšiny, Polná, and Hroby. Trade routes also existed between Bavaria (Germany) and South Bohemia for trading salt (Praxl 1993), but after 1706 the salt trade on these routes was forbidden by the Habsburg Empire (Praxl 1993; Hajná 2011). In addition, transport on these pathways was difficult because of the steepness of the terrain. Thus, the road from Upper Austria to Bohemia (Linzer Steig) became the main trade route for salt and cattle.

Another reason for the strong differentiation of the German populations is found in their population history. Annual census data from 1989 to 1998 provide evidence of severe demographic bottlenecks in all German populations and evidence for local extinctions (Rösler 2001). Although the populations at Mauth and Sonnen have recovered in terms of numbers in recent years (Zipp 2009; Dolek et al. 2010), the demographic bottlenecks are still evident in the patterns of genetic diversity. Hence, our results provide another example where recent population history, rather than current conditions, determines genetic structure (see e.g., Xu et al. 2006; Jaramillo and Atkinson 2011).

### Temporal structure

The very low differentiation between the two cohorts (2007/2008) of the population at Chvalšiny (Czech Republic) is in strong contrast to the high differentiation found between the 2007 and 2008 cohorts of the three German populations. This might be due to reduced seed bank activation by cattle hoofprints after a period of abandonment in the German populations, while the Czech population has experienced a much longer period of traditional grazing with additional regular soil disturbance through military training activities in recent decades (A. Pavlíčko, pers. comm.). Traditional land use was practiced much longer and more intensely in this part of Bohemia than in Bavaria, where abandonment and afforestation started before the Second World War (Breuer et al. 2010).

Without seed bank activation, the biennial life cycle of *G. bohemica* results in temporal isolation among consecutive cohorts. Acyclic activation of the persistent seed bank (Brabec 2010b; J. Brabec, unpubl. data), however, can favor mating between the descendants of different cohorts. Such activation can be promoted by patchy disturbance of soil and vegetation by intensive grazing, through temporally irregular management methods, or due to annual variation in weather conditions. Stadler et al. (2010) assumed that differential activation of the seed bank was the reason for the appearance of a second gene pool in a large structured population of *G. aspera*, occurring in a viticultural landscape with varying management. There have been several reports of disturbance opening the soil, allowing the seed bank to be activated, and thus allowing *G. bohemica* seedlings to be successfully recruited (Rösler 2001; Brabec 2005; Engleder 2006).

### Second gene pool in Mauth 2008

Ten of 16 analyzed individuals from Mauth 2008 did not belong to the gene pool including all other individuals collected in both years in Mauth and Finsterau as revealed by the STRUCTURE analysis. Instead, they were included in the large Czech and Austrian group, from which they may be separated by combining the first and third PCoA axes. In the field, these individuals were not growing separately or spatially clustered according to the detected genetic groups. The genetic pattern without admixture in the individuals of the second gene pool indicates recent seed dispersal. We consider pollen dispersal an unlikely source of the patterns of genetic differentiation for two reasons: (1) Pollen dispersal only carries a haploid genome and is expected to result in a pattern of significant admixture, which we did not observe. (2) Pollen dispersal is considered the major component of gene flow at the local scale, that is, within populations (Ouborg et al. 1999). In addition, interpopulation pollen dispersal is unlikely given the spatial separation from the Czech and Austrian populations.

Thus, the genetic profile most likely indicates that these individuals were migrants or descendants of migrants from other Czech or Austrian populations that were not included in this analysis. The mixed mating system (i.e., selfing and outcrossing) found in *G. bohemica* (Brabec 2010a) and in other taxa of the genus *Gentianella* (Fischer and Matthies 1997; Wagner and Mitterhofer 1998; Greimler and Dobeš 2000) probably helps to conserve genetic structure. Given high selfing rates, descendants of migrants may retain the genetic characteristics of their source population for several generations without admixture from the population in which they are found. Another reason for the lack of admixture may be that the migrant seeds have been buried in the seed bank for some time. Occasional long-distance dispersal of *Gentianella* seeds may have happened in the past for example, along trade routes (see above) by sheep and cattle, as such small seeds can become lodged in sheep's wool (Fischer et al. 1996; Tackenberg et al. 2006) and are also dispersed to a high degree endozoochorically (Brunn and Poschlod 2006).

In our study, we were able to identify a possible source area (although not a single source population) for those migrants. It was, however, impossible to determine dispersal time and vectors from the present data. To our knowledge, conservation activities such as seed sowing in declining German populations have always used seeds from the same or nearby populations (Götz 1993; Rösler 2002; S. Rösler and T. Zipp, pers. comm.), but earlier activities might not have been documented. Seed dispersal between Mauth and Finsterau may have been facilitated by mowing and grazing the two sites with the same animals or machines in the early 1990s (T. Zipp, pers. comm.). Metapopulation dynamics in a strict sense (Freckleton and Watkinson 2002), however, are unlikely on a regional scale in *Gentianella* species. Such dynamics may act on a local scale among patches of large spatially structured populations (facilitated by management and animal vectors) as suggested by Stadler et al. (2010) for *G. aspera*.

### Diversity within populations

Our data confirm that the effective population size *N*_e_ is a better predictor of genetic diversity (*H*_Sh_) than the current population size (Ellstrand and Elram 1993). For species with large year-to-year fluctuations in numbers of individuals, the effective population size is best estimated as the harmonic mean of the population sizes of several consecutive years (Frankham 1996), where data are available. For instance, a severe demographic bottleneck (only five individuals in year 2004) occurred in the population at Onšovice, which showed a high number (>1100) of individuals in 2008. The effective population size was strongly reduced due to this event and this is reflected in the low levels of genetic diversity. Overall, we also observed relationships between genetic diversity and size-related plant traits.

Annual censuses can be complicated by seasonal dimorphism, as observed in many *Gentianella* taxa (e.g., Wettstein 1896; Lennartsson 1997). However, such temporal separation within the same population and year has become very rare in Central Europe (Skalický 1969; Zopfi 1991; V. Skalický, pers. comm.) and has not been observed in the populations involved in this study (Procházka 2001; Engleder 2006; J. Königer, pers. obs.). A single strong decline of the population size in one year can also be buffered by a persistent seed bank that becomes activated after the bottleneck.

The low genetic diversity found in the German populations may result both from inbreeding and genetic drift effects in years with very small numbers of individuals. At neutral loci, bottlenecks have a stronger effect on allelic richness than on heterozygosity (Widmer and Lexer 2001), while inbreeding essentially reduces the latter. The AFLP fragment numbers (*n*_F_) we found were inconclusive with respect to recent bottlenecks. A codominant marker would be needed to estimate allelic variation and heterozygosity.

### Conclusions

The genetic structure of the investigated populations of *G. bohemica* is largely explained by earlier periods of connectivity between Czech and Austrian populations. Many populations have experienced recent demographic bottlenecks, which have affected their effective population size and genetic diversity. In the long term, the poor dispersal ability of plants limited to rare and isolated habitats is expected to result in strong genetic differentiation among populations (Givnish 2010). Several of our studied populations may already be trapped in one of the extinction vortices suggested by Gilpin and Soule (1986). Given a persistent seed bank, dynamics of remnant populations (Silvertown and Charlesworth 2001), that is, recruitment from the seed bank, may buffer high demographic stochasticity and bottlenecks. Those dynamics, however, are currently poorly understood and such observations should be included in conservation and monitoring programs for endangered species.

## Supporting Information

**Appendix S1.** Census data (number of flowering plants) from regional monitoring programs with census periods ranging from seven to 20 consecutive years.

**Appendix S2.** Summary of the STRUCTURE analysis of AFLP data.
