# Supplementary material for: Spatial and temporal determinants of genetic structure in Gentianella bohemica
Source: Ecol Evol. 2012 Mar;2(3):636–48. doi: 10.1002/ece3.211 (PMC3399150; doi:10.1002/ece3.211)
Supplement: Supplementary file 1 [file ece30002-0636-SD1.doc]

**Appendix 2.** Census data (number of flowering plants) from regional monitoring programs with census periods ranging from seven to 20 consecutive years.

|  | **Finsterau** | **Mauth** | **Sonnen** | **Chvalšiny** | **Hroby** | **Onšovice** | **Polná** | **Aigen** | **Leopoldschlag** |
| --- | --- | --- | --- | --- | --- | --- | --- | --- | --- |
|  |  |  |  |  |  |  |  |  |  |
| 1989 | 1 | 211 | 0 |  |  |  |  |  |  |
| 1990 | 11 | 162 | 40 |  |  |  |  |  |  |
| 1991 | 4 | 156 | 19 |  |  |  |  |  |  |
| 1992 | 0 | 163 | 860 |  |  |  |  |  |  |
| 1993 | 0 | 174 | 182 |  |  |  |  | 60 |  |
| 1994 | 3 | 165 | 193 |  |  |  |  | 50 |  |
| 1995 | 2 | 74 | 16 |  |  |  |  | 60 |  |
| 1996 | 5 | 14 | 11 |  |  |  |  | 75 |  |
| 1997 | 5 | 64 | 14 |  |  |  |  | 38 |  |
| 1998 | 6 | 76 | 45 |  |  |  |  | 45 |  |
| 1999 | 0 | 78 | 32 | 300 | 591 | 500 |  | 80 |  |
| 2000 | 24 | 285 | 38 | 270 | 520 | 3021 |  | 53 |  |
| 2001 | 1 | 71 | 184 | 512 | 514 | 1000 |  | 61 |  |
| 2002 | 35 | 105 | 125 | 1134 | 700 | 452 | 5000 | 30 | 112 |
| 2003 | 2 | 18 | 88 | 590 | 63 | 85 | 103 | 49 | 12 |
| 2004 | 3 | 34 | 40 | 219 | 76 | 5 | 151 | 32 | 132 |
| 2005 | 14 | 118 | 299 | 1566 | 3057 | 783 | 7750 | 97 | 715 |
| 2006 | 24 | 25 | 126 | 2735 | 426 | 946 | 1043 | 95 | 165 |
| 2007 | 30 | 420 | 368 | 230 | 2820 | 643 | 918 | 121 | 950 |
| 2008 | 16 | 92 | 271 | 1360 | 1055 | 1108 | 937 | 318 | 365 |
