# Supplementary figures and images for: Spatial and temporal determinants of genetic structure in Gentianella bohemica
Source: Ecol Evol. 2012 Mar;2(3):636–48. doi: 10.1002/ece3.211 (PMC3399150; doi:10.1002/ece3.211)

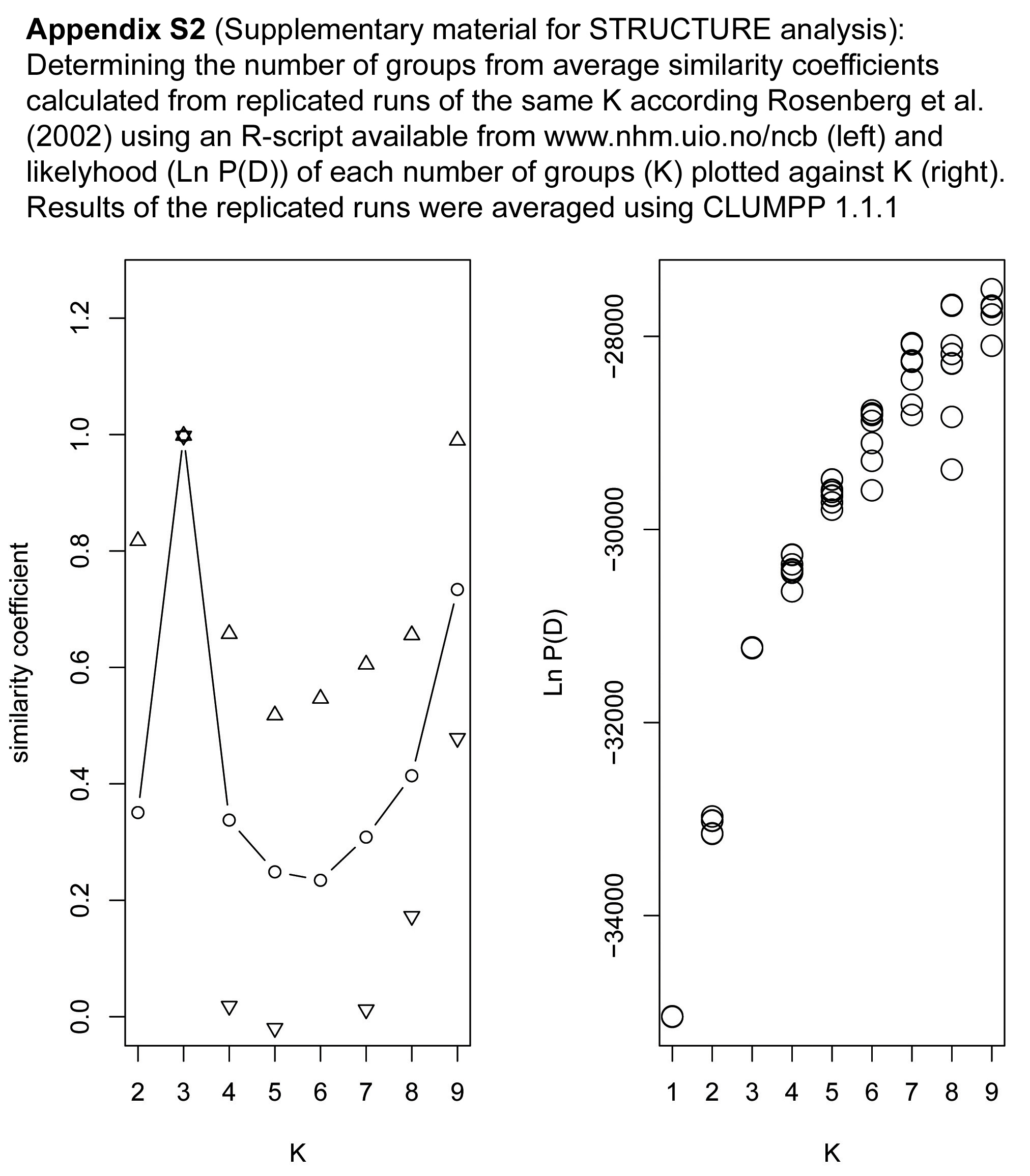

Supplement: Supplementary file 2 [file ece30002-0636-SD2.jpg]
